# [^177^Lu]Lu-DOTA-TATE versus standard of care in adult patients with gastro-enteropancreatic neuroendocrine tumours (GEP-NETs): a cost-consequence analysis from an Italian hospital perspective

**DOI:** 10.1007/s00259-021-05656-x

**Published:** 2021-12-24

**Authors:** Francesca Spada, Davide Campana, Giuseppe Lamberti, Riccardo Laudicella, Renato Dellamano, Luca Dellamano, Oscar Leeuwenkamp, Sergio Baldari

**Affiliations:** 1 Division of Gastrointestinal Medical Oncology and Neuroendocrine Tumors, European Institute of Oncology (IEO), IRCCS, Milan, Italy; 2 Division of Medical Oncology, IRCCS Azienda Ospedaliero-Universitaria di Bologna, Via P. Albertoni 15, 40138 Bologna, Italy; 3 Nuclear Medicine Unit, Department of Biomedical and Dental Sciences and of Morpho-Functional Imaging, University of Messina, Viale Consolare Valeria n.1, Messina, Italy; 4 ValueVector, Milan, Italy; 5 Advanced Accelerator Applications/A Novartis Company, Geneva, Switzerland

**Keywords:** Cost-consequence analysis; Everolimus, Gastro-enteropancreatic neuroendocrine tumour, Healthcare costs, Italy, Lutetium, Peptide receptor radionuclide therapy, Progression-free survival, Somatostatin receptor agonists, Sunitinib

## Abstract

**Purpose:**

To assess and compare clinical outcomes and costs, to the Italian healthcare system, of three therapeutic options approved in the management of adult patients with gastro-enteropancreatic neuroendocrine tumours (GEP-NETs).

**Methods:**

We compared the efficacy, safety, and costs of [177Lu]Lu-DOTA-TATE, everolimus (both originator and generic products), and sunitinib in patients with advanced GEP-NETs (NET G1 and G2) that had progressed following treatment with somatostatin analogs (SSAs). A cost-consequence model was developed and validated by a panel of clinical experts from three NET reference centres in Italy. The clinical outcomes included in the model were median progression-free survival and the incidence of grade 3 or 4 adverse events (AEs), as reported in pivotal clinical trials. The costs for acquisition and administration of each treatment, and of managing AEs, were calculated from the perspective of the Italian national health service. Treatment costs per progression-free month were calculated separately for patients with NETs of pancreatic (PanNETs; all three treatments) and gastrointestinal (GI-NETs; [177Lu]Lu-DOTA-TATE and everolimus only) origin.

**Results:**

In patients with PanNETs, total costs per progression-free month were €2989 for [177Lu]Lu-DOTA-TATE, €4975 for originator everolimus, €3472 for generic everolimus, and €5337 for sunitinib. In patients with GI-NETs, total costs per progression-free month were €3189 for [177Lu]Lu-DOTA-TATE, €4990 for originator everolimus, and €3483 for generic everolimus.

**Conclusions:**

[177Lu]Lu-DOTA-TATE was associated with lower costs per progression-free month versus relevant treatment options in patients with GI-NETs or PanNETs (NET G1–G2; progressed following SSA treatment), although acquisition and administration costs are higher. These findings provide further economic arguments in the overall context of treatment decision-making.

**Supplementary Information:**

The online version contains supplementary material available at 10.1007/s00259-021-05656-x.

## Introduction

Neuroendocrine neoplasms (NENs), better known as neuroendocrine tumours (NETs), arise from the diffuse neuroendocrine system, which is distributed throughout the human body. Thus, NETs can originate in any part of the body, but most commonly arise in the gastro-enteropancreatic (GEP) tract. Indeed, GEP-NETs accounted for 3.56 cases per 100 000 in the Surveillance, Epidemiology, and End Results (SEER) 18 registry [1]. Even though they are still considered rare, the incidence of NETs has risen markedly in recent decades. For example, in the USA, it increased from 1.09 to 6.98 per 100 000 population between 1973 and 2012, representing a 6.4-fold increase [1]. In the UK, the age-standardised incidence rate of GEP-NETs in 2013–2015 has been reported as 4.6 per 100 000 [2]. In Italy, there were an estimated 2700 new cases of NETs in 2015, of which 1242 (46%) were GEP-NETs; the crude rate of GEP-NETs for 2000–2010 was approximately 1.9 per 100 000 [3].

Histologically, NENs can be divided into two main groups: well-differentiated tumours (NETs) and poorly differentiated neuroendocrine carcinomas (NECs). Additionally, NETs can be divided into four categories (NET G1, NET G2, NET G3, and NEC) depending on morphology and proliferation index (Ki-67); each has distinct clinical and biomolecular features and prognostic features in terms of survival [4].

Early-stage GEP-NETs are often asymptomatic, or present with poorly defined symptoms [5]. Consequently, these neoplasms are usually diagnosed at an advanced stage [5], at which point a significant percentage of patients will have metastases (primarily in the liver). Typically, clinical management takes a multimodal approach (i.e. surgery, embolisation, radiotherapy, and medical treatment). Several systemic treatments are available for the management of patients with locally advanced or metastatic NETs, including chemotherapy and interferon alfa-2b [6], somatostatin analogs [7, 8], everolimus [9, 10], and, for pancreatic NETs (PanNETs) only, sunitinib [11]. Somatostatin analogs (SSAs) have long been a mainstay of treatment for NETs, particularly for patients with functioning tumours who require symptomatic relief [12]. Peptide receptor radionuclide therapies such as [177Lu]Lu-DOTA-TATE (Lutathera®) have also entered clinical practice [13–15]. Among these treatments, only everolimus, sunitinib, and [177Lu]Lu-DOTA-TATE are currently approved for the treatment of progressive GEP-NETs. The specific indication for [177Lu]Lu-DOTA-TATE is the treatment of unresectable or metastatic, progressive, well-differentiated, somatostatin receptor–positive GEP-NETs in adults [14]; it has orphan drug designation in both the USA and Europe [16, 17].

In the phase III NETTER-1 trial, [177Lu]Lu-DOTA-TATE significantly lowered the risk of disease progression in patients with inoperable, progressive midgut NETs [13]. At the time of the data cutoff for the primary analysis (at month 20: July 24, 2015), the median progression-free survival (PFS) was 8.4 months for high-dose octreotide long-acting release (LAR), but was not reached for [177Lu]Lu-DOTA-TATE (hazard ratio [HR], 0.21; 95% confidence interval, 0.13–0.33; *P* < 0.001). In addition, the estimated risk of death was 60% lower with [177Lu]Lu-DOTA-TATE versus octreotide LAR (HR, 0.4; *P* = 0.004) [13]. Subsequently, cost-effectiveness analyses of [177Lu]Lu-DOTA-TATE for the treatment of GEP-NETs have been undertaken in support of reimbursement applications in several countries.

In the absence of relevant data from randomised trials, the optimum sequence for the use of systemic treatments for GEP-NETs is unknown. However, one meta-analysis submitted in conjunction with a cost-effectiveness model to the National Centre for Health and Care Excellence (NICE) in the UK suggested a survival benefit of [177Lu]Lu-DOTA-TATE compared with current care [18]. NICE provided a positive recommendation for [177Lu]Lu-DOTA-TATE in patients with gastrointestinal (GI)-NETs or PanNETs, on the basis of demonstrated cost-effectiveness [19]. The economic model submitted to NICE for their evaluation was a partitioned survival model with pre-progression, post-progression, and death states. In the post-progression state, the overall survival (OS) benefit of [177Lu]Lu-DOTA-TATE unfavourably impacted the cost-effectiveness analysis [20].

In Italy and other countries, there is a need for a simpler model that is based on published PFS data and includes the costs of circumventing the unfavourable impact of prolonged OS. Moreover, research into biological and clinical predictors of response is still ongoing, and the profile of patients most likely to respond to each option is therefore unknown. In this context, a pharmacoeconomic model, designed to assess and compare the clinical outcomes and costs associated with competing therapeutic options, may be a useful aid to decision-making based on survival and cost aspects.

Accordingly, we developed a specific novel cost-consequence model to evaluate treatment options, in clinical and economic terms, for Italian patients with advanced GEP-NETs (NET G1 and NET G2; progressed following SSA therapy).

## Materials and methods

A cost-consequence model was designed to compare the clinical outcomes (efficacy and safety) and costs associated with three approved treatment alternatives ([177Lu]Lu-DOTA-TATE, everolimus, and sunitinib) for patients with advanced GEP-NETs (NET G1 and NET G2; progressed following SSA therapy) in Italy. The main metric of this study was the cost of each treatment option per progression-free month, taking into account the costs of drug acquisition, administration, and toxicity.

Median PFS data for patients with advanced (unresectable or metastatic) PanNETs or GI-NETs were taken from the official summaries of product characteristics (SmPCs) [14, 21, 22] and were consistent with the key results of pivotal trials of each treatment option published in peer-reviewed journals [9–11, 13, 15, 23] and utilised by the NICE in their assessment of the same therapies [19]. Median PFS, defined as the time from randomisation to progressive disease or death, was chosen for several reasons. Firstly, it was the primary efficacy endpoint in each trial [9–11, 13, 15, 23], and secondly, it was considered to most accurately reflect the survival benefit of [177Lu]Lu-DOTA-TATE, particularly in patients with progressive disease. In contrast to median OS, median PFS is not confounded by post-progression treatment, including treatment crossover. Thus, median PFS is a more direct reflection of underlying antitumour efficacy, and consequently, cost per progression-free month is a relatively unbiased outcome measure. Thirdly, analyses based on median PFS are straightforward and not confounded by the accrual of costs over a longer duration during the post-progression state. Lastly, at the time of developing the present model, median OS for [177Lu]Lu-DOTA-TATE was not yet known, precluding its use in the model.

Model assumptions on the efficacy [177Lu]Lu-DOTA-TATE in GI-NETs were based on the results of the phase III NETTER-1 trial [13, 14]. This was a randomised, active-controlled comparison of [177Lu]Lu-DOTA-TATE, plus best supportive care (BSC; octreotide LAR 30 mg every 28 days for symptom control), versus high-dose (60 mg) octreotide LAR every 28 days in patients with progressive, somatostatin receptor–positive, advanced midgut NETs. Of the 231 patients recruited, 117 received [177Lu]Lu-DOTA-TATE plus BSC and 114 received high-dose octreotide LAR. The groups were balanced with respect to tumour grade, somatostatin receptor expression, and previous treatment [13]. For PanNETs, median PFS for [177Lu]Lu-DOTA-TATE was obtained from the single-arm, monocentric, phase I/II Erasmus study [14, 15].

Median PFS data for everolimus and sunitinib were based on the results of randomised phase III placebo-controlled trials in which patients received BSC in addition to assigned study treatment. The RADIANT-3 and RADIANT-4 trials compared everolimus with placebo in patients with PanNETs and GI-NETs, respectively [9, 10], while another trial (NCT00428597) compared sunitinib with placebo in patients with PanNETs [11, 23].

### Safety

The main measure of safety used in the model was the incidence of grade 3 or 4 adverse events (AEs), applying the National Cancer Institute Common Terminology Criteria for Adverse Events (CTCAE) that were in use at the time each study was conducted. Data were abstracted from SmPCs [14, 21, 22]; however, because SmPCs report AEs by category, and not by severity, we also used AE data from original clinical trial publications for each therapeutic option [9–11, 13, 15, 23]. Data were validated by five of the study investigators (FS, DC, GL, RL, and SB).

### Costs: base-case scenario

Costs were calculated from the perspective of the Italian national health service (NHS) over a time horizon of 1 year and are reported in 2020 euros (€) [24–26].

For [177Lu]Lu-DOTA-TATE, acquisition costs were calculated for a complete course of therapy, defined according to the approved posology for the product and consisting of a total of up to four intravenous infusions of 7400 MBq each [14]. The analysis was conservative because, on average, patients in the NETTER-1 trial received 3.55 doses instead of the 4 doses recommended in the approved SmPC [13]. For everolimus (both generic and originator products) and sunitinib, acquisition costs were calculated for 1 year of continuous therapy at the relevant approved dosage (everolimus: 10 mg/day orally; sunitinib: 37.5 mg/day orally) [21, 22] and assumed that adherence was 100%. This approach reflects the structure and design of the respective Agenzia Italiana del Farmaco (AIFA) registries created for the management of these drugs in Italy and takes into account the periodic efficacy assessments that are required for ongoing reimbursement.

Patients with NETs routinely receive treatment with an SSA for the management of symptoms associated with functional tumours. Therefore, the acquisition costs of supportive treatment with SSAs were calculated for 1 year’s treatment for all three therapies. In the case of everolimus and sunitinib, costs were based on an ‘average’ dose of long-acting SSAs (octreotide LAR: 30 mg every 28 days; lanreotide: 120 mg every 28 days) and an assumed adherence rate of 100%. For [177Lu]Lu-DOTA-TATE, these costs were calculated as the sum of long-acting SSAs costs for 36 weeks and costs of short-acting SSAs for each 4-week period (for a total of 12 weeks) prior to treatment, in line with the SmPC [14] and the design of the NETTER-1 trial.

Additionally, [177Lu]Lu-DOTA-TATE must be co-administered with an intravenous amino acid infusion to reduce the radiation dose absorbed by the kidneys and, consequently, the risk of renal toxicity (see Supplementary Appendix 1 in Online Resource 1) [14]. In clinical trials, some amino acid products were associated with nausea and vomiting that necessitated the use of anti-emetic treatment [13]. Therefore, costs were included in the model for both amino acid and anti-emetic therapy. It was conservatively assumed that all patients would require such treatment; however, the amino acid infusion recommended by the manufacturer of [177Lu]Lu-DOTA-TATE (see Supplementary Appendix 1 in Online Resource 1) causes nausea and vomiting at a much lower rate. Given the relatively low costs of such therapies, it was also assumed that these costs were included in the basic national tariff associated with diagnosis-related group (DRG) 409 (see below) [25].

#### Drug administration

[177Lu]Lu-DOTA-TATE can only be administered in a designated nuclear medicine facility by healthcare professionals authorised to handle radiopharmaceuticals [14]. Although current Italian legislation (Art. 158 of Legislative Decree 101/2020^1^) does not mandate that patients receiving lutetium-based radiopharmaceuticals are treated as in patients, we conservatively assumed that each dose of [177Lu]Lu-DOTA-TATE would require hospitalisation for at least 1 day. Hospitalisation costs for [177Lu]Lu-DOTA-TATE were calculated using the National Tariff for Hospital Services for Acute Patients, DRG 409 [25]. Everolimus and sunitinib are administered orally, and therefore, no administration costs related to overnight hospitalisation were assigned to either drug in the model.

#### Follow-up

The follow-up strategy for low-grade NENs is similar for patients receiving [177Lu]Lu-DOTA-TATE, everolimus, or sunitinib.^2^ Thus, we assumed that follow-up costs would be the same for each treatment option and excluded them from the model.

#### Management of adverse events

To our knowledge, no published data exist regarding the management modalities of adverse events of therapies used for the treatment of patients with PanNETs and GI-NETs and the associated costs. In the absence of such data, the reasonable approach that is normally accepted in the context of recently introduced health technologies is to use estimates generated by experts’ opinions. For this purpose, several remote working sessions were held between March and May 2020. These were attended by clinical experts from three Italian centres responsible for the care of a large number of NET patients. The experts (FS, DC, GL, RL, and SB) had extensive experience in the treatment of NETs considered reflective of current clinical practice in Italy. Hospital admission frequencies were based on clinical practice at these centres. Estimates used in the model were based on consensus among the clinical experts.

The estimated frequencies of resources used for the management of adverse events (specialists’ visits and hospital admissions, ≥ 1 or < 1 day) were subsequently converted into costs using the tariffs associated with the relevant ambulatory and hospital services.

In particular, to calculate the costs associated with specialist appointments and/or hospitalisations resulting from grade 3 or 4 AEs, current tariffs for specialist ambulatory services and for DRG-related hospital services were used. For ambulatory service costs, the most recent versions of the National Tariff for Hospital Services for Acute Patients [25] and the Regional Tariff for Hospital Services for Lombardy (the Nomenclatore Tariffario di Regione Lombardia) [24] were used.

The base-case assumption was that any grade 3 or 4 AE would prompt at least one consultation at a specialist centre. The cost of these visits was calculated as the mean of the tariffs in the National [25] and Regional Tariff for Hospital Services (Lombardy) [24]. We also assumed that, in practice, a specialist visit would lead to hospital admission, at least in a certain percentage of patients, either for a standard in-patient stay (≥ 1 day) or for day care (< 1 day). For each treatment, we identified the DRG codes associated with the management of each of the grade 3 or 4 AEs documented in phase III clinical trials [9–11, 13] and calculated the costs for both types of hospitalisation.

### Sensitivity analyses

The purpose of sensitivity analysis is to investigate the degree to which the conclusions are sensitive to changes in the assumptions that underpin the model. However, sensitivity analyses tend to be arbitrary and may not reflect reality; for example, drug acquisition costs used in the base-case analysis are official prices minus mandatory discounts, but discounts in real-world practice may be greater. Thus, instead of presenting our own sensitivity analyses, conducted by adjusting key variables by predetermined amounts or percentages, we developed a simple, flexible tool that allows healthcare professionals and payers in Italy to input data relevant to their own institutions; this allows immediate generation of institution-specific cost-consequence data for each treatment option.

Given the flexibility of the model, several simulations were performed to address specific questions of potential interest to real-world users in the context of the Italian NHS. Following discussion in an ad hoc working session, the authors identified the following questions, based on expected changes in drug acquisition costs, real-world experience with regard to variance in dose exposure and adherence, and potential changes in hospitalisation modalities for patients receiving [177Lu]Lu-DOTA-TATE:

1. What discount would be needed for everolimus (original brand and/or generic) and sunitinib to match the modelled costs per progression-free month obtained in the base-case simulation for [177Lu]Lu-DOTA-TATE?
2. How would the modelled costs per progression-free month change if the dose intensity or adherence assumed for the three treatments were lower than 100%?
3. How would the modelled costs per progression-free month change if patients receiving [177Lu]Lu-DOTA-TATE were treated in a day-hospital setting, instead of being hospitalised for at least 1 day? In this case, a reduced tariff of €353 for DRG 409 was applied.

In each simulation, all other variables were kept constant, in order to explore the impact of each specific factor on the end result.

## Results

### Efficacy

For [177Lu]Lu-DOTA-TATE, the median PFS used in the model was 28.4 months in patients with GI-NETs and 30.3 months in patients with PanNETs [14]. Median PFS was 11.0 months for everolimus (for both GI-NETs and PanNETs) [21] and 11.4 months for sunitinib (PanNETs only) [22].^3^

### Safety

In total, 26 different grade 3 or 4 AEs were identified and mapped to DRG hospital codes (Table 1). The resource use associated with grade 3 or 4 AEs, as determined by a clinical expert validation exercise, is presented in Table 2. Safety data were not available for patients with PanNETs who received [177Lu]Lu-DOTA-TATE in the Erasmus phase I/II study, so the available data for GI-NETs from NETTER-1 were extrapolated to both indications.

**Table 1:** Drug-related grade 3 or 4 adverse events with ≥ 1% incidence in phase III trials of [177Lu]Lu-DOTA-TATE, everolimus, and sunitinib in the treatment of pancreatic and gastrointestinal neuroendocrine tumours (PanNETs and GI-NETs, respectively) [9–11, 13]

**Adverse event, *n* (%)**	**[177Lu]Lu-DOTA-TATE** (*n* = 111) [13]^a^	**Everolimus** [9, 10]		**Sunitinib** [11]
		PanNETs (*n* = 204)	GI-NETs (*n* = 202)^b^	PanNETs (*n* = 83)
Abdominal pain	3 (3)	–	–	4 (5)
Anaemia	–	12 (6)	8 (4)	–
Anorexia/decreased appetite	–	–	1 (< 1)	2 (2)
Diarrhoea	3 (3)	7 (3)	15 (7)	4 (5)
Dyspnoea	–	–	2 (1)	–
Epistaxis	–	–	–	1 (1)
Fatigue or asthenia	2 (2)	–	–	8 (10)
Fatigue	–	5 (2)	7 (3)	4 (5)
Asthenia	–	2 (1)	3 (1)	4 (5)
Flushing	1 (1)	–	–	–
Hair colour changes	–	–	–	1 (1)
Hyperglycaemia	–	11 (5)	7 (3)	–
Hypertension	–	–	–	8 (10)
Infections^c^	–	5 (2)	14 (7)	–
Leucopenia	1 (1)	–	–	–
Lymphopenia	10 (9)	–	–	–
Mucosal inflammation	–	–	–	1 (1)
Musculoskeletal pain	2 (2)	–	–	–
Nausea^d^	4 (4)	5 (2)	3 (1)	1 (1)
Neutropenia	1 (1)	–	–	10 (12)
Palmar-plantar erythrodysesthesia	–	–	–	5 (6)
Peripheral oedema	–	1 (< 1)	4 (2)	–
Pneumonitis^e^	–	5 (2)	3 (1)	–
Pyrexia	–	–	4 (2)	–
Stomatitis^f^	–	14 (7)	18 (9)	3 (4)
Thrombocytopenia	2 (2)	8 (4)	–	3 (4)
Vomiting^d^	8 (7)	–	–	–
Weight loss	–	–	–	1 (1)

All data are expressed as n (%)

^a^Due to the lack of detailed information on grade 3 or 4 adverse events in patients with PanNETs receiving [177Lu]Lu-DOTA-TATE, data obtained in patients with GI-NETs (from the NETTER-1 trial [13]) were extrapolated to patients with PanNETs

^b^Data are from the RADIANT-4 trial [9], which included patients with lung NETs as well as GI-NETs

^c^All types of infections are included

^d^Episodes caused by co-administration of amino acid infusion are excluded

^e^Includes interstitial lung disease, lung infiltration, and pulmonary fibrosis

^f^Includes aphthous stomatitis, mouth ulceration, and tongue ulceration

*GI-NET* gastrointestinal neuroendocrine tumours, *PanNET* pancreatic neuroendocrine tumour

**Table 2:** Resource use associated with drug-related grade 3 or 4 adverse events (AEs)

Grade 3 or 4 AE (% of total)	Initial assessment visit	No hospitalisation	Day hospital (< 1 day)	Standard hospitalisation (> 1 day)
Anaemia	100	0	92	8
Anorexia	100	50	50	0
Flushing	100	100	0	0
Asthenia/fatigue	100	100	0	0
Hair colour changes	100	100	0	0
Diarrhoea	100	0	50	50
Dyspnoea	100	0	50	50
Abdominal pain	100	10	90	0
Musculoskeletal pain	100	100	0	0
Peripheral oedema	100	100	0	0
Epistaxis	100	50	50	0
Palmar/plantar erythrodysesthesia	100	100	0	0
Mucosal inflammation	100	50	30	20
Infections	100	100	0	0
Hyperglycaemia	100	0	90	10
Hypertension	100	95	0	5
Leukopenia	100	95	0	5
Lymphopenia	100	100	0	0
Nausea^a^	100	100	0	0
Neutropenia	100	95	0	5
Weight loss	100	100	0	0
Pyrexia	100	100	0	0
Pneumonitis	100	0	50	50
Stomatitis	100	50	30	20
Thrombocytopenia	100	0	90	10
Vomiting^a^	100	50	50	0

^a^Nausea and vomiting episodes caused by co-administration of amino acids (65% of nausea episodes and 73% of vomiting episodes) were excluded from the calculation, since the related costs were already included in the hospital diagnosis-related group tariff for administration of the drug

With the exception of haematological AEs, the incidence of individual grade 3 or 4 AEs in the NETTER-1 trial was similar for the active and control groups [13]. Haematological events were transient, and there was no evidence of renal toxicity among patients who received [177Lu]Lu-DOTA-TATE co-administered with an amino acid infusion over a median follow-up period of 14 months [13].

### Costs

#### Base-case analysis

Costs associated with each treatment option, and modelled costs per progression-free month, calculated in the base-case scenario, are shown in Table 3. Treatment with [177Lu]Lu-DOTA-TATE was associated with the lowest cost per progression-free month in patients with PanNETs and GI-NETs, despite having the highest costs for drug acquisition and administration. In the treatment of PanNETs, the cost of [177Lu]Lu-DOTA-TATE per progression-free month (€2989) was 40% less than for originator everolimus (€4975), 14% less than for generic everolimus (€3472), and 44% less than for sunitinib (€5337). In the treatment of GI-NETs, the cost of [177Lu]Lu-DOTA-TATE per progression-free month was €3189, which was 36% less than for originator everolimus (€4990) and 8% less than for generic everolimus (€3483), assuming (conservatively) that all four doses of [177Lu]Lu-DOTA-TATE were administered.

**Table 3:** Treatment costs (in 2020 euros [€]) over 12 months

Cost element	[177Lu]Lu-DOTA-TATE	Everolimus		Sunitinib
		Originator	Generic	
**Costs per 12 months (€)**				
Drug acquisition^a^	74 000	41 587	24 995	47 517
Drug administration		NA	NA	NA
If hospitalisation < 1 day	1412	NA	NA	NA
If hospitalisation > 1 day (base case)	5884	NA	NA	NA
Supportive treatment with SSA	10 635	13 184	13 184	13 184
**PanNETs**				
Management of AEs (€)	54	156	156	137
Total cost (€) (base case)	90 519	54 772	38 180	60 701
Median PFS (months)	30.3	11.0	11.0	11.4
Total cost per month of PFS (€)	2989	4975	3472	5337
**GI-NETs**				
Management of AEs (€)	54	168	168	NA
Total cost (€)	90 519	54 772	38 180	
Median PFS (months)	28.4	11.0	11.0	
Total cost per month of PFS (€)	3189	4990	3483	

^a^Costs for study drug

*AE* adverse event, *GI-NET* gastrointestinal neuroendocrine tumours, *NA* not applicable, *PanNET* pancreatic neuroendocrine tumour, *PFS* progression-free survival, *SSA* somatostatin analogs

#### Sensitivity analyses

In PanNETs, the purchase-price discounts required to match the base-case cost per progression-free month obtained for [177Lu]Lu-DOTA-TATE were calculated to be 52.7% for originator everolimus, 21.4% for generic everolimus, and 56.3% for sunitinib. For GI-NETs, the discounts required were 47.7% for originator everolimus and 13.0% for generic everolimus.

We also explored the effects of variations in dose intensity and adherence. Assuming that dose intensity for [177Lu]Lu-DOTA-TATE was equal to the actual mean number of doses in NETTER-1 (i.e. 3.55 doses) and that adherence to everolimus and sunitinib was 85%, costs per progression-free month in the treatment of PanNETs were €2687 for [177Lu]Lu-DOTA-TATE, €4231 for originator everolimus, €2954 for generic everolimus, and €4538 for sunitinib. In GI-NETs, costs were €2867 for [177Lu]Lu-DOTA-TATE, €4244 for originator everolimus, and €2963 for generic everolimus.

Adjusting dose intensity/adherence for all products to 75%, costs per progression-free month in the treatment of PanNETs were €2318 for [177Lu]Lu-DOTA-TATE, €3735 for originator everolimus, €2608 for generic everolimus, and €4006 for sunitinib for PanNETs. For GI-NETs, costs per progression-free month were €2473 for [177Lu]Lu-DOTA-TATE, €3746 for originator everolimus, and €2616 for generic everolimus.

Lastly, changing the length of hospitalisation for [177Lu]Lu-DOTA-TATE administration to < 1 day reduced the cost of this treatment option (per month of PFS) to €2842 in PanNETs and to €3032 in GI-NETs.

Overall, the results were most sensitive to variations in drug acquisition costs and adherence rates. Variations in the costs of managing AEs did not markedly influence the results (data not shown).

## Discussion

Economic evaluation has become an important step in the integration of new technologies into medical practice and forms the basis of reimbursement decisions in countries (including Italy) where healthcare resources are finite. Such evaluation usually measures the costs and consequences of the new technology against the existing standard of care and enumerates its incremental cost per unit of benefit gained. Essentially, the choice of a specific methodological approach depends on the kind of decision that the analysis is intended to inform: the greater the complexity of the decision (e.g. national reimbursement decisions on new health technologies), the more complex the methodological approach will be.

In Italy, [177Lu]Lu-DOTA-TATE, everolimus, and sunitinib are recognised as innovative therapies and, accordingly, are reimbursed by the Italian NHS for GI-NETs and/or PanNETs [27–32]; this permits formulary inclusion and prescribing (in accordance with the respective approved labelling for each product) at a regional level. However, the final decision to use an innovative therapy lies with hospitals and healthcare providers, and it is therefore important to provide straightforward, simple, transparent, and methodologically appropriate economic data that play a supportive role in an informed decision-making process.

Our cost-consequence model was developed with these considerations in mind: the intervention of interest ([177Lu]Lu-DOTA-TATE) was compared with two alternative treatments in terms of costs per progression-free month; published median PFS data and grade 3/4 AEs were included in the model because these are the variables with the greatest influence on treatment selection in patients with GEP-NETs. Everolimus (indicated for both PanNETs and GI-NETs) and sunitinib (indicated for PanNETs only) were included in the analysis because they are the only approved alternatives to [177Lu]Lu-DOTA-TATE for progressive GEP-NETs (NET G1-G2, progressed following SSA therapy) in Italy; high-dose octreotide LAR is not approved in these indications and therefore was not included in the analysis.

We found that [177Lu]Lu-DOTA-TATE had higher acquisition and treatment costs than sunitinib or everolimus but resulted in markedly longer median PFS than either of the oral agents. In both the base-case and sensitivity analyses, this translated into lower costs per progression-free month versus comparators.

We believe that our study is the first pharmacoeconomic comparison of systemic treatments for GEP-NETs in Italy. Economic analyses of [177Lu]Lu-DOTA-TATE have been reported in conjunction with reimbursement dossiers. In one analysis, conducted from the perspective of the French healthcare system, [177Lu]Lu-DOTA-TATE was associated with incremental costs of €42 106 per quality-adjusted life-year (QALY) gained versus high-dose octreotide LAR and €59 769 per QALY gained versus everolimus in the treatment of advanced GI-NETs [33]. The authors concluded that [177Lu]Lu-DOTA-TATE was likely to be considered cost-effective in France on the basis of these results but noted that the findings were sensitive to the survival data used. In England and Wales, NICE has issued a positive recommendation for [177Lu]Lu-DOTA-TATE in patients with GI-NETs and PanNETs [19], and the drug is also fully reimbursed in Scotland [34].

Our model used efficacy and safety data from well-controlled clinical trials. Currently, real-world information regarding [177Lu]Lu-DOTA-TATE in the treatment of GEP-NETs is limited, although a study is planned in Italy. In the Erasmus phase I/II study, patients with bronchial NETs or GEP-NETs who received ≥ 22.2 GBq of [177Lu]Lu-DOTA-TATE (*n* = 443) had an overall median PFS of 29 months [15]. In another Dutch study of 168 patients with advanced bronchial NETs or GEP-NETs who required salvage therapy with [177Lu]Lu-DOTA-TATE, median PFS was 35.4 months after initial treatment, 14.6 months after retreatment, and 14.2 months after a third treatment with [177Lu]Lu-DOTA-TATE [35].

In a prospective observational study of [177Lu]Lu-DOTA-TATE in Sweden, 200 patients with a broad range of advanced NETs had a median PFS of 27 months; however, patients in whom the absorbed dose to the kidneys was < 23 Gy had a median PFS of only 15 months, compared with 33 months in those receiving ≥ 23 Gy [36]. This is relevant to our study because we assumed adherence rates of 100% for all treatments, including [177Lu]Lu-DOTA-TATE. In clinical practice, serious or severe haematological, renal, and other toxicities may necessitate temporary dosage reduction, treatment interruption, or permanent discontinuation [14, 37]; these events can limit the total delivered dose and thus reduce the expected clinical benefits of treatment. In the NETTER-1 trial, the average number of doses administered per patient was 3.55 and not 4 as conservatively assumed in our model (i.e. highest possible cost assuming administration of all planned doses) [13].

To address this issue, sensitivity analyses were performed using dose intensities or adherence rates < 100% for each treatment option. All of the scenarios tested resulted in lower costs per progression-free month for [177Lu]Lu-DOTA-TATE versus everolimus (originator and generic) and/or sunitinib in both indications.

We also investigated the level of purchase-price discount that would be required, in both PanNETs and GI-NETs, for everolimus and/or sunitinib to match the base-case cost of [177Lu]Lu-DOTA-TATE per progression-free month. In the treatment of PanNETs, discounts of > 50% were needed for originator everolimus and sunitinib, whereas reductions of > 20% were needed for generic everolimus. In GI-NETs, discounts of 48% for originator everolimus and 13% for generic everolimus would be required.

The costs of [177Lu]Lu-DOTA-TATE per progression-free month were further reduced if treatment was administered in a day-hospital instead of an in-patient setting. Our model was specifically designed so that users could input costs for the duration of hospitalisation relevant to their institution or jurisdiction, reflecting either a conservative (≥ 1 day of hospitalisation) or non-conservative (< 1 day of hospitalisation) scenario.

Another consideration is that the hospital costs associated with administration of [177Lu]Lu-DOTA-TATE in a day-hospital setting were quantified using the national tariff [25], resulting in an additional cost of €353 per dose. Depending on the region, this tariff may be up to 90% lower if [177Lu]Lu-DOTA-TATE is included in the regional list of separately funded high-cost drugs (the so-called ‘File F’ list). Thus, hospital costs for this treatment option (as well as overall costs) would be lower in regions where [177Lu]Lu-DOTA-TATE is included in the ‘File F’ list than in the scenario we have presented.

We did not adjust our results for the effects of treatment on health-related quality of life (QoL). In the NETTER-1 trial, [177Lu]Lu-DOTA-TATE was associated with a statistically and clinically significant QoL benefit (i.e. a significantly longer time to deterioration of EORTC QLQ-C30 and G.I.NET-21 scores) versus high-dose octreotide [38]. Furthermore, significant improvements in several aspects of the global health score (i.e. physical/role/emotional/social functioning scales) have been also reported [39, 40]. In contrast, no QoL advantage over placebo was observed for either everolimus [41] despite a significant reduction in physical functioning scale [42] or for sunitinib in the NCT00428597 trial [43] despite significant worsening in insomnia and diarrhoea scores compared to placebo in Pan NET [43]. The lack of head-to-head trials means that it is not possible to directly compare the effects of [177Lu]Lu-DOTA-TATE, everolimus, and sunitinib on QoL in patients with GEP-NETs; nevertheless, the data from trials versus octreotide and placebo are interesting and may help to guide decision-making. The inclusion of QoL and OS in a future iteration of the model is being considered.

Patient preference is increasingly important in medical decision-making and is a complex construct resulting from the interplay between drug (i.e. efficacy, tolerability, route of administration) and patient-specific factors (i.e. beliefs, values, socioeconomic status, location, lifestyle). It is a separate area of study and, although highly relevant, has not yet been included in our model.

One possibly contentious assumption used in our model was that the costs of follow-up would be the same for [177Lu]Lu-DOTA-TATE as for everolimus and sunitinib. It could be argued that exposure to [177Lu]Lu-DOTA-TATE might pose an increased risk of long-term renal or haematological complications relative to everolimus or sunitinib, which may necessitate a closer follow-up of the patient. Follow-up assumptions made in the model were based on published evidence and consensus of Italian experts reflecting current clinical practice in Italy. Further, a long-term safety analysis of the NETTER-1 study revealed a benign safety profile with no new safety signals of any kind [44]. The long-term tolerability of ^90^Y and/or ^177^Lu peptide receptor radionuclide therapy in a large series (*n* = 807) of patients with NETs treated at the European Institute of Oncology in Milan can be regarded as supportive [45]. Thus, we believe that the patient follow-up as applied in the model is sensible, justifiable, and reflective of current clinical practice in Italy. The model was primarily based on the NETTER-1 study, in which patients who received [177Lu]Lu-DOTA-TATE had a mean age of 63 years [13], characteristic for these patients when treated with this treatment modality. The older age of the patient population provides further support for the potentially conservative follow-up assumption in the model as [177Lu]Lu-DOTA-TATE’s safety profile is seemingly favourable relative to that of the comparators in the model. In addition, it is noteworthy that model outcome was driven by benefit in terms of progression-free survival as shown by sensitivity analyses.

Lastly, the results of our analysis reflect the perspective of the Italian NHS, and the experience of only three centres (albeit expert centres) within it, and cannot necessarily be applied *tout court* to other settings without prior customisation. The estimated costs associated with the management of adverse events of the three therapeutic approaches under evaluation should be further validated through detailed research and analysis of real-world data from clinical practice in the Italian healthcare setting, such as NCT04727723 (Real-Lu) for [177Lu]Lu-DOTA-TATE. However, considering the relatively low frequency of grade 3–4 adverse events for all three therapies, we do not anticipate this to significantly affect the end results of the overall analysis.

In conclusion, from the perspective of the Italian NHS, [177Lu]Lu-DOTA-TATE was associated with lower costs per progression-free month than either everolimus or sunitinib in the treatment of advanced GEP-NETs, by virtue of its markedly longer median PFS, according to the results of our cost-consequence model that included AE-related costs. This model may help to guide informed treatment choices in patients with advanced GEP-NETs based on clinical and economic parameters.

## Supplementary Information

Below is the link to the electronic supplementary material.

Supplementary file1 (DOCX 44 KB)

## Data Availability

Data sharing is not applicable to this article as no datasets were generated or analysed during the current study.

## Footnotes

1 Legislative Decree no.101/2020 reorganised the sector legislation, establishing safety standards in order to protect people from the risks associated with exposure to ionising radiation in the industrial, medical, and research environments. It also regulates the safety of nuclear plants, installations, and activities in which radioactive materials are used, as well as the management of spent fuel and radioactive waste.

2 Includes biochemical and morphological imaging (computed tomography [CT], magnetic resonance imaging [MRI], and/or functional imaging (positron emission tomography [PET] with DOTA-peptides).

3 Updated PFS data for sunitinib have been published (Faivre S, et al. *Ann Oncol* 2017;28(2):339–343) but, at the time of writing (March 2021), do not yet appear in the SmPC for the product. For consistency, we used PFS data validated by the EMA and included in the SmPC for all three treatments.
