# Supplementary material for: [177Lu]Lu-DOTA-TATE versus standard of care in adult patients with gastro-enteropancreatic neuroendocrine tumours (GEP-NETs): a cost-consequence analysis from an Italian hospital perspective
Source: Eur J Nucl Med Mol Imaging. 2021 Dec 24;49(6):2037–48. doi: 10.1007/s00259-021-05656-x (PMC9016001; doi:10.1007/s00259-021-05656-x)
Supplement: Supplementary file 1 — Supplementary file1 (DOCX 44 KB) [file 259_2021_5656_MOESM1_ESM.docx]

**LUTETIUM [^177^Lu] OXODOTREOTIDE VERSUS STANDARD OF CARE IN ADULT PATIENTS WITH GASTRO-ENTEROPANCREATIC NEUROENDOCRINE TUMOURS (GEP-NETs): A COST-CONSEQUENCE ANALYSIS FROM AN ITALIAN HOSPITAL PERSPECTIVE**

**Authors:** Francesca Spada,^1^ Davide Campana,^2^ Giuseppe Lamberti,^2^ Riccardo Laudicella,^3^ Renato Dellamano,^4^ Luca Dellamano,^4^ Oscar Leeuwenkamp,^5^ Sergio Baldari^3^

**Affiliations:**

**1** Division of Gastrointestinal Medical Oncology and Neuroendocrine Tumors, European Institute of Oncology, IRCCS, Milan, Italy

**2** IRCCS Azienda Ospedaliero-Universitaria di Bologna, Bologna, Italy

**3** Nuclear Medicine Unit, Department of Biomedical and Dental Sciences and of Morpho-Functional Imaging, University of Messina, Messina, Italy

**4** ValueVector, Milan, Italy

**5** Advanced Accelerator Applications/A Novartis company, Geneva, Switzerland

**Corresponding author:**

Professor Sergio Baldari

Nuclear Medicine Unit, Department of Biomedical and Dental Sciences and of Morpho-Functional Imaging, University of Messina

Viale Consolare Valeria n.1, Messina, Italy

Tel: +390902212840

Email: sergio.baldari@unime.it

**Journal:** *European Journal of Nuclear Medicine and Molecular Imaging*

## Supplementary Appendix 1. Intravenous amino acid solution

An amino acid solution must be infused intravenously with each dose of lutetium [^177^Lu] oxodotreotide, for renal protection purposes [15]. The infusion should begin 30 minutes before the start of lutetium [^177^Lu] oxodotreotide and should last for 4 hours.

The amino acid solution can be prepared as a compounded product, complying with good practice regarding the preparation of sterile medicinal products, and according to the composition specified in Table SA1.

**Table SA1.** Composition of the standard amino acid solution

| **Compound** | **Amount** |
| --- | --- |
| Lysine (g) | 25 |
| Arginine (g) | 25 |
| Sodium chloride 9 mg/mL (0.9%) solution for injection (L) | 1 |

Alternatively, some commercially available amino acid solutions can be used if compliant with the specification described in Table SA2. However, the compounded solution has a lower total infusion volume and osmolarity than commercially available solutions, and is therefore considered the product of choice.

**Table SA2.** Specification of commercially available amino acid solutions

| **Characteristic** | **Specification** |
| --- | --- |
| Lysine content (g) | 18–24 |
| Arginine content (g) | 18–24 |
| Volume (L) | 1.5–2.2 |
| Osmolarity (mOsmol/L) | <1050 |
